# Navigating the Garden Path: Evidence for Restructuring as a Shared Process

**DOI:** 10.3390/jintelligence14070128

**Published:** 2026-07-01

**Authors:** Sarah K. C. Dygert, Andrew F. Jarosz

**Affiliations:** Department of Psychology, Mississippi State University, Starkville, MS 39762, USA; skc195@msstate.edu

**Keywords:** creativity, problem-solving, ambiguity, comprehension

## Abstract

Resolving misrepresentations is key when faced with ambiguous information. For example, problem-solvers may misrepresent the constraints of a problem, while readers may misrepresent parts of a sentence. This work investigates how a shared cognitive process might facilitate the restructuring of representations during complex tasks. Students from Mississippi State University participated in studies demonstrating that similar restructuring processes are necessary for successful creative problem-solving and garden path sentence comprehension. Experiment I demonstrated that successful creative problem-solving correlates with successful garden path sentence resolution after removing the variance of non-ambiguous sentence comprehension. Experiment II demonstrated that successful garden path sentence comprehension predicts creative problem-solving, even after controlling for working memory capacity and fluid intelligence. Furthermore, this relationship was unique to creative problem-solving, as garden path comprehension had no bearing on analytic problem-solving success. Results are taken as evidence for a shared cognitive process that aids in revising misrepresented information across certain contexts. This work provides an understanding of how individuals grapple with inconsistencies between the goals of their tasks and their representations of the world.

## 1. Restructuring and Problem-Solving

The Gestalt psychologists’ (Köhler, 1959; Ohlsson, 1984) studies of insightful, or creative, problem solving suggests that constructing an appropriate problem representation is paramount for efficient problem solving, with inappropriate representations leading to impasse. Successfully reaching a solution requires something beyond normal problem-solving processes: “restructuring” the problem space to remove the impasse. If these restructuring processes truly underlie creative problem solving, then it is possible that processes akin to restructuring may also underlie performance differences in other complex task environments in which misrepresentation occurs. The current set of studies tests whether restructuring is also related to the revisionary processes involved in ambiguous language comprehension (but not non-ambiguous language comprehension). Such a finding would demonstrate the validity and relevance of the process of restructuring as critical for creative (but not analytic) problem solving, as well as its role beyond the specific context of problem solving. Broadly, the need to restructure (and subsequently, individual differences in the likelihood to *effectively* restructure) is discussed as a context- and resource-dependent process that becomes prevalent when routine processing fails.

Theories differ about the particular processes and mechanisms that underlie creative versus non-creative solutions in problem solving (Chuderski & Jastrze̜bski, 2018; Knoblich et al., 1999; Kounios & Beeman, 2014; Ohlsson, 1984; Seifert et al., 1995; Smith, 1995; Weisberg, 2006), though a common theme emerges among many of them: the need to shift or restructure an inappropriate problem representation into a sufficient one. Problem solvers initially build a mental representation (Knoblich et al., 1999; Newell & Simon, 1972; Ohlsson, 1984), then use familiar algorithms or heuristics to reach a solution (Ash & Wiley, 2006; Beilock & DeCaro, 2007; Wiley & Jarosz, 2012). In many cases, these approaches result in solving the problem; however, for some problems, algorithms and heuristics may fail to produce a solution. In this case, solvers may find themselves at an “impasse,” rendering them unsure of how to continue. This period of interrupted problem solving is when restructuring of the insufficient initial representation likely occurs (Cranford & Moss, 2012; DeCaro et al., 2016; Köhler, 1959; Ohlsson, 1984; Wiley & Jarosz, 2012; but see Kounios & Beeman, 2014, for evidence of insight without impasse or restructuring). Restructuring then leads to a solution and “insight,” in which the solution appears suddenly without prior awareness of solution progress. Theoretically, restructuring has been associated with relaxing unnecessary constraints or decomposing “chunks” of information (Knoblich et al., 1999); forgetting unhelpful/fixated information (Smith, 1995); activating remote ideas in memory through spreading activation (Bowden & Beeman, 1998; Mednick, 1962; Ohlsson, 1984); retrieving/satisfying open goals (Moss et al., 2007, 2011; Seifert et al., 1995); or otherwise revising the faulty representation.

Restructuring is a latent process that is generally indirectly measured from subjective reports of insight and/or from binary accuracy rates across a set of problems varying in difficulty (see Cushen & Wiley, 2012; Danek & Wiley, 2017; Kounios & Beeman, 2014 for promising direct measures). Some theorists have argued against restructuring-based theories of creativity, suggesting instead that “creative” solutions are simply epiphenomena of routine processes operating in different ways to produce unique effects (Chuderski & Jastrze̜bski, 2018; Perkins, 1981; Weisberg, 2006). However, considerable evidence suggests that routine processes cannot fully explain the phenomenon of insight or the solutions that often accompany it (Ansburg & Hill, 2003; Ash & Wiley, 2006; Benedek et al., 2017; Dygert & Jarosz, 2020; Jarosz et al., 2012; Moss et al., 2007).

Research on the role of restructuring in problem solving often contrasts problems that are best solved via analytic solution methods with creative problems (DeCaro et al., 2016; Wiley & Jarosz, 2012). “Analytic” problems (e.g., algebra problems, Tower of Hanoi; Simon, 1975) are reliably solved by incremental methods (e.g., heuristics/algorithms; Newell & Simon, 1972); “creative” problems, in contrast, are presumed to be novel to participants and are susceptible to misrepresentation, thus requiring restructuring processes. Successful restructuring often results in spontaneous insight and unique solutions (Ohlsson, 1984). As opposed to analytic problems, solvers are metacognitively unaware of solution progress when solving creative problems (Metcalfe, 1986; Metcalfe & Wiebe, 1987), benefitting from “use your gut” strategies as opposed to incremental or algorithmic methods (Aiello et al., 2012). Ultimately, findings such as these suggest that these problem classifications, based on presumed novelty to participants, are useful for experimentally differentiating between unique or shared processes across analytic and creative solution methods.

Past work investigating individual differences in problem solving has suggested that restructuring lies below the threshold of awareness (Metcalfe & Wiebe, 1987). Even subjective insight ratings, though commonly used as a measure of insight (and potentially restructuring), are limited in their ability to adequately represent the spontaneous processing that precedes the feeling of insight. Some researchers interpret insight ratings as an indicator of the use of creative processes in reaching a solution (Bowden & Jung-Beeman, 2003; Cushen & Wiley, 2012), while others interpret insight ratings as merely an epiphenomenon of mundane processes behaving in unexpected ways (Chuderski & Jastrze̜bski, 2018; Weisberg, 2006). This theoretical nuance has also contributed to ongoing debates about the influence of routine/default processes such as working memory capacity (WMC), fluid intelligence (gF), or special processes (i.e., restructuring) in promoting creative versus non-creative solutions.

One way of resolving these disagreements is through an individual-differences approach. If creative problem solving is driven by WMC or gF, then those constructs should explain considerable variance in creative problem solving. A failure to do so would suggest that alternative processes must be driving solution performance. A dual-process account walks a middle ground, suggesting that attention-loading constructs (e.g., WMC; gF) are only beneficial for exhausting the faulty search space, after which attention-loading constructs such as WMC and gF do not aid, and potentially harm, solution progress (Aiello et al., 2012; Ash et al., 2009; Ash & Wiley, 2006; DeCaro, 2018; Ricks et al., 2007; DeCaro et al., 2016; Wiley, 1998). Another study used structural equation modeling to compare theoretically driven models involving the relationship between WMC and creative cognition (Dygert & Jarosz, 2020), in which results demonstrated that while WMC explained considerable domain-specific variance, it only explained 2% of the variance shared across creativity tasks. Though it is unclear what the largely unexplained domain-general variance represents, a restructuring mechanism seems a plausible explanation. Notably, such restructuring processes are unlikely to be problem-solving specific.

## 2. Restructuring and Language Comprehension

In this vein, creative problem solving is not likely the only area of cognition in which restructuring is relevant. For example, in language comprehension, individuals often develop meaningful interpretations of words and phrases before the entire context has been revealed. In garden path sentences, structurally- and/or semantically ambiguous units are initially misinterpreted, rendering an incomplete understanding that requires revisions. According to the “good-enough” approach to sentence comprehension (Christianson et al., 2001; Ferreira & Patson, 2007; Qian et al., 2018), the initial misinterpretation occurs because readers represent the simplest possible structure of the sentence, only modifying this representation when absolutely necessary. For example, readers often assume that, “*When the actor performs the play containing profanities runs late and people leave,*” maintains a subject-verb-object structure, suggesting that “*the play*” is the object of “*the actor performs.*” They then attach new incoming information (i.e., “*containing profanities*”) to material currently being processed, instead of attaching or separating material elsewhere. Readers then attempt to minimize the distance of separated information to acquire the simplest possible path to comprehension. When comprehending normal, non-ambiguous language, these processes lead to a complete understanding of the sentence. When interpreting locally ambiguous language, however, these processes mislead readers to attach/separate information incorrectly. In the latter case, some readers may be left with an incohesive and incomplete understanding, which may compel them to reevaluate and modify the faulty interpretation depending on its severity. By reinterpreting “*When the actor performs*” as a subject-verb structure instead of a subject-verb-object structure, readers can correctly re-interpret the sentence as depicting the actor’s performance as a separate event from the play containing profanities.

Just as with creative problem solving, multiple studies have reported minimal effects of WMC on the ability to correctly resolve a locally ambiguous sentence (e.g., Von der Malsburg & Vasishth, 2013; Waters & Caplan, 1996) when compared to the general benefit of higher WMC on non-ambiguous sentence comprehension (Daneman & Carpenter, 1980). For example, higher-WMC individuals have a greater tendency than their lower-span counterparts to overcommit to their initial representation of a sentence, leading to increased susceptibility to misrepresenting garden path sentences (Von der Malsburg & Vasishth, 2013). Other work found that differences in WMC also did not correlate with performance differences (i.e., error rates) in comprehension for garden path sentences nor of control sentences (Waters & Caplan, 1996). Interestingly, individuals lower in WMC are more likely to attach information at a more distant site in the sentence rather than at a more local site; in contrast, individuals higher in WMC tend to prefer the opposite (Swets et al., 2007), lending support to the idea that individual differences in WMC can impact reading strategies and subsequently, successful comprehension. Likewise, older adults tend to endorse incomplete interpretations of ambiguous language when compared to their younger counterparts, perhaps due to a greater need to preserve WMC resources in older adults (Christianson et al., 2006). Together, these studies re-iterate a nuanced relationship between WMC and the comprehension of both normal and ambiguous language, such that re-analysis of ambiguous sentences cannot be deemed reliant on WMC-based resources.

Given these similarities, interpreting garden path sentences may utilize similar processes as those in creative problem-solving to correctly resolve ambiguities. In both cases, processing begins with an incorrect representation, followed by individuals using their learned methods to acquire the simplest paths to the solution. This leads to the individual getting stuck, fixating on their initial faulty representation and attempting to restructure that representation. Both creative solvers and readers may initially use a simple heuristic to strategically reach a solution/understanding of the sentence. In both instances, individuals will utilize these familiar, incremental-type methods until there are no more paths available from the initial faulty representation, suggesting that impasse or incomplete comprehension has been reached. With all forward paths exhausted, the initial representation must be restructured to one including the solution or correct understanding. It is the overlap of these last two processes that are the focus of the present work.

## 3. Present Research

The present work tests for a common restructuring process in problem solving and language comprehension. Several hypotheses were tested: (1) If successfully interpreting garden path sentences relies on restructuring processes similar to those recruited during creative problem-solving tasks, then success in creative problem solving will correlate with success in garden path sentence comprehension. (2) This correlation will persist even when partialling out the variance explained by non-ambiguous sentence processing. (3) The shared variance between creative problem solving and garden path sentence processing will not be explained by differences in WMC and gF. (4) There will not be a relationship between analytic problem-solving success and garden path sentence processing.

In Experiment I, sentence type was manipulated to test whether ambiguous sentence comprehension relates to solution success on problems that tend toward creative solutions, above and beyond what can be explained by non-ambiguous sentence comprehension. Notably, no “creative” problem is guaranteed to be solved via creative processes and many are solvable via creative or analytic processes (Bowden & Beeman, 1998; Kounios & Beeman, 2014; Salvi et al., 2015), with the nuances of the particular problem likely driving the impact of individual differences on problem-solving approaches (Ash et al., 2009; Ash & Wiley, 2006; DeCaro et al., 2016). However, evidence suggests that certain classes of problems tend to be solved via creative processes, while others are almost always solved via analysis (Ash & Wiley, 2008; Metcalfe & Wiebe, 1987). As such, Experiment II uses problems that tend toward either analytic solution processes or creative solution processes, while also controlling for individual differences in WMC and gF as theoretically driven constructs of interest.

Rebus problems (in Experiment I) and Anagrams (in Experiments I and II) were used as measures likely to require restructuring. In looking at the obstacles to solving these problems (as suggested by Ash et al., 2009), these problems lack a clear algorithm or heuristic that leads to an efficient solution. In addition, anagrams could suffer from fixation on frequent bigrams (e.g., the letters “c” and “h” appearing in sequence), while Rebus problems require focus on non-linguistic information (such as spatial location on the page). In both cases, restructuring may be necessary. In contrast, tasks such as modular arithmetic can always be solved algorithmically. Thus, while the present work does not contain direct measures of restructuring, it taps into this latent construct by examining shared and unique variance between tasks.

### 3.1. Experiment I

#### 3.1.1. Method

**Participants.** Eighty-five undergraduate students (25 males, 60 females) enrolled in General Psychology at Mississippi State University volunteered to participate in this study as one of several options through which to receive partial course credit. Participation was completed outside of class time in the authors’ research lab in groups of one to four at a time. The number of participants was selected via a power analysis using G*power version 3.1.9.2 (Faul et al., 2009) for a power of 0.80 to find a medium-to-large correlation (Cohen, 1992), and represented a convenience sample. Because each task used in this study was heavily dependent on English syntax and semantics, all participants were also native English speakers. This study was reviewed and approved by the Mississippi State University IRB. All participants volunteered and provided written consent to participate.

**Materials.** All tasks were completed using desktop computers. Participants completed three tasks, which (when combined) took approximately one hour: a garden path sentences task, an anagram task, and a Rebus puzzle task. Tasks were presented in a randomized order for each participant, and the items within each task were also randomized. Detailed accounts of each task are provided below.

***The Garden Path Task.*** The garden path task used modified materials and procedures from Ferreira and Henderson (1991). The task embedded locally ambiguous garden path sentences among a set of non-ambiguous control sentences. The task prompted participants to carefully read and interpret each sentence and then judge whether a truth-value statement was “true” or “false” as a measure of sentence comprehension (see Table 1 for garden path task examples).

Participants viewed four example sentences and 50 experimental sentences divided between two sentence types (ambiguous and non-ambiguous). All sentences in the task followed a basic structure: a dependent clause followed by an independent clause, and no comma between them. Twelve of the 50 sentences were locally (temporarily) ambiguous garden path sentences (i.e., grammatically correct, yet misleading sentences). Each garden path sentence contained a dependent clause ending with a verb and an independent clause beginning with a noun phrase. This structure was intended to be the source of local ambiguity, resulting in misinterpretations of the noun phrase as the direct object of the preceding verb.

The remaining 38 sentences in this task were non-ambiguous, grammatically correct sentences. These non-ambiguous control items followed the same basic structure mentioned above: a dependent clause followed by an independent clause, and no comma between them. The control sentences’ dependent clauses ended with either a noun phrase or a prepositional phrase. This structure was intended to comply with readers’ expected pattern of constituent separation, resulting in correct initial interpretations of these sentences.

“Yes/no” comprehension questions may unintentionally reveal a sentence’s intended meaning (rather than the reader’s true interpretation) due to the presence of the memory probe itself, which may challenge participants’ lingering misrepresentations and result in spontaneous revisions to the original interpretation that otherwise may not have occurred (Ferreira & Patson, 2007). Thus, truth-value statements were used to avoid biasing participants to inferentially or logically guess correct answers or the purpose of the study (Gordon, 1998), as well as to avoid biasing participants towards affirming the question with “yes” rather than rejecting it with “no” (Johnson, 1987; Knowles & Condon, 1999). Truth-value statements require participants to respond “true” or “false” to a comprehension statement, rather than a question, and were used to assess how participants interpreted each sentence. The truth-value statements for the garden path sentences (12) and the control items (38) were divided equally between true and false statements. Two versions of the garden path task were created to counterbalance true and false truth-value statements for each sentence.

Before the task began, participants reviewed written instructions explaining the general procedure of the task. The instructions also notified participants that all of the items in the task were grammatically correct, English sentences. This precaution was taken to ensure that participants did not automatically respond with “false” truth-value judgments when they believed a sentence to simply be ungrammatical (i.e., misinterpreted garden path sentences). After reviewing the instructions, participants provided truth-value judgments for four example items (two ambiguous, two control). For practice items, participants read one sentence at a time, and they pressed the spacebar after carefully reading and understanding each item. The next slide displayed a truth-value statement that referred to the preceding sentence. Participants pressed “T” on the keyboard if the statement was true and “F” if the statement was false. After providing a judgment, the next slide displayed the correct answer. The practice procedure was intended to acclimate the participants to the structure of the task, including the truth-value statements and the types of sentences. Participants then viewed and responded to the experimental items in the same manner as the practice items described above, without feedback. Participants continued this process until all 50 sentences had been read and truth-value responses were collected. Participants were scored according to the proportion of accurate responses to truth-value statements for both ambiguous and control sentences.

***The Anagram Task*.** The anagram task used modified materials from Gilhooly (1978). Anagrams are a type of creative problem in which the letters of a particular word or phrase are scrambled and must be rearranged to uncover that word or phrase (e.g., ONEGM → GNOME). The task included 30 five-letter, one-solution anagrams covering a range of difficulty (based on Gilhooly’s bigram rank calculations), with the letters in each anagram presented in a fixed, randomized order. Post-task experimental analyses revealed that one anagram contained a typo, resulting in 29 usable anagrams. Three additional anagrams were used as example items. Participants completed the three example problems before starting the experimental portion. After providing a response for each example item, the correct answer was presented; correct answers were not provided for the experimental items. Each anagram was shown one at a time for a maximum of 30 s before the problem timed out. Participants indicated when they had solved each anagram by immediately pressing the spacebar. Participants had a maximum of 10 s to enter their solution into the free-response box on the next slide and press “Enter” to view the next problem. Scores consisted of the proportion of correctly solved anagrams.

**Figure 1:**
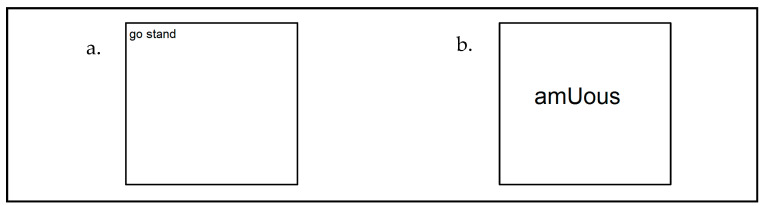
Rebus puzzle task examples: (**a**) Go stand in the corner; (**b**) Ambiguous.

***The Rebus Puzzle Task*.** This task used modified materials and methods from MacGregor and Cunningham (2008) and followed a similar procedure as the anagram task. Rebus puzzles are a type of creative problem that requires a solver to utilize and combine visual, verbal, and spatial clues in the problem to uncover a common idiom or phrase. Figure 1a depicts an easier Rebus problem with the solution, “Go Stand in the Corner,” which can be reached by simply interpreting the phrase “go stand” as being in the corner of the problem box. On the other hand, Figure 1b represents the more difficult solution “Ambiguous,” which requires the solver to first interpret that the size of the capital letter “U” is “big,” and then also phonetically incorporate the “big U” as part of the entire word.

Participants attempted to solve each problem and responded with the correct phrase that each Rebus puzzle described. A total of 24 experimental Rebus problems and three practice problems covering a range of difficulty were used. Following MacGregor and Cunningham’s (2008) procedure, each Rebus puzzle was presented one at a time, and participants had 30 s to solve each problem before it timed out. Participants indicated if they solved the puzzle before the end of the time limit by pressing the spacebar. The next slide prompted them to type their solution into a free-response box within a 20 s time limit. Once they provided a response, participants pressed the “Enter” key to move on to the next problem. Scores on the Rebus task were computed as the proportion of correct responses given during the task.

**Procedure.** Participants were seated at a computer in a testing room. After providing written informed consent, the experimenter gave general instructions about each of the three tasks. Because task order was randomized for each participant, the experimenter loaded and started each of the tasks for every participant. As each participant completed the experiment, they were thanked, debriefed, and dismissed.

**Transparency and Openness.** Neither of the experiments reported in this article were formally preregistered. The data from both studies is available on the Open Science Framework at: https://osf.io/a87k6/overview?view_only=fca6773a1c834df68cc544f1d81c9d9e (accessed on 4 April 2026). The materials used in these studies are widely available, and are available from the authors upon request.

#### 3.1.2. Results

Table 2 shows descriptive statistics of overall task accuracy and Cronbach’s alpha, and Table 3 shows task correlations. Scores on the anagram and Rebus tasks significantly correlated with each other, as well as with the resolution of garden path sentences. Notably, reliability of garden path sentence accuracy was somewhat low (α = 0.62), which may suppress correlations with that variable.

Analyzing individual tasks risks an increased impact of task-specific effects on results. Utilizing a composite score reduces the influence of such effects, better isolating the variance of interest. A composite creativity score was calculated by first computing the *z*-score for each creativity task, and then averaging those scores together. As seen in Table 3, the composite creativity scores positively correlated with correct interpretations of garden path sentences and with correct interpretations of control sentences. A partial correlation between the composite creativity scores and correctly interpreted garden path sentences, controlling for non-ambiguous control sentence comprehension, showed a positive relationship between creativity scores and garden path sentence interpretations, *r* = 0.33, *p* = .002. This suggests 11% of the variance is shared between these constructs (R^2^ = 0.11), a moderate effect size in psychological research. In contrast, a partial correlation between creativity scores and control sentence accuracy controlling for garden path sentence accuracy showed no relationship, *r* = 0.14, *p* = .215. Repeating these analyses with the creativity variables independently yielded identical results, with the exception that the correlation between garden path sentences and anagram accuracy was only marginally significant when controlling for control sentence accuracy, *r* = 0.21, *p* = .06.

As frequentist statistics present issues with interpreting null results, a Bayesian regression was calculated, with a null model containing control sentence accuracy predicting composite creativity scores, and a comparison model including ambiguous sentence accuracy. The model including ambiguous sentence accuracy had 19.59 times more evidence supporting it than a model with only control sentence accuracy (BF_10_ = 19.59). In contrast, placing ambiguous sentence accuracy in the null model and then comparing a model with control sentence accuracy added suggested the data are twice as likely to occur under the null model than the model with both predictors (BF_10_ = 0.49).

**Exploratory Analyses.** No a priori predictions were made regarding response time; however, one might expect a similar pattern of results if restructuring increases the difficulty of both sentence processing and creative problem-solving. Thus, analyses were repeated using response time instead of accuracy as the variables of interest. Results replicated accuracy analyses: A partial correlation between the composite creativity response time and correctly interpreted garden path sentence reading time, controlling for non-ambiguous control sentence reading time, showed a positive relationship between creativity and garden path sentence reading, *r* = 0.23, *p* = .04. This suggests 5% of the variance is shared between these constructs (R^2^ = 0.05), a small effect. In contrast, a partial correlation between creativity response times and control sentence reading time controlling for garden path sentence accuracy showed no relationship, *r* = 0.00, *p* = .99.

#### 3.1.3. Discussion

This preliminary investigation examined evidence for shared processes between creative problem solving and garden path sentence comprehension. Results demonstrated that successful creative problem solving was indeed related to successful garden path sentence comprehension, even after controlling for success at normal sentence comprehension. This was further supported by strong evidence from the Bayesian analysis, suggesting almost 20 times the evidence in favor of the alternative hypothesis over the null. Furthermore, there was no relationship between non-ambiguous sentence comprehension and creative problem solving after accounting for garden path sentence interpretations. Each of these results was echoed in an exploratory response time analysis. These results suggest a potential shared restructuring mechanism underlying both abilities. Per theories of null hypothesis testing, the absence of a finding does not provide evidence against that finding; though there was no significant relationship between control sentences and problem solving this does not prove that a relationship is not there. That said, the Bayesian analysis provided a BF_10_ of 0.49, suggesting twice as much evidence in favor of the null hypothesis over the alternative. This provides weak evidence that there is indeed no relationship and is somewhat inconclusive. One limitation is the lower reliability of the garden path sentence accuracy measure, which may suppress correlations. Further work is therefore warranted. Experiment II explores this finding further while addressing some of the limitations of Experiment I.

### 3.2. Experiment II

One alternative interpretation of the results in Experiment I is attributing these relationships to known correlates of problem solving and language comprehension, such as WMC or gF (Chuderski & Jastrze̜bski, 2018; Daneman & Carpenter, 1980; Dygert & Jarosz, 2020; Turner & Engle, 1989). However, results are mixed concerning the role of WMC in creative problem solving (Wiley & Jarosz, 2012), with considerable work demonstrating a nonexistent, or even negative, relationship between the two (DeCaro et al., 2016; Dygert & Jarosz, 2020). Furthermore, WMC may aid in the initial processing of problems, but not in the act of restructuring itself (Ash & Wiley, 2006). Experiment II addresses these possibilities directly by including measures of WMC and gF. Ambiguous sentence comprehension should again bear a unique relationship with creative problem solving after accounting for variability in these measures. Finally, an analytic problem solving condition was included to demonstrate that restructuring is unique to creative, but not routine, problem solving. Garden path resolution should not relate to analytic problem-solving ability any more than non-ambiguous sentence comprehension, as the solution to analytic problems should, in general, require no restructuring.

Another test of shared processes draws upon the fact that other types of ambiguous sentences may not require restructuring. For example, sentences with two viable interpretations may be ambiguous, but do not require restructuring for successful comprehension. Consider the sentence, “*The grandmother of the heiress who bankrupted herself last year still made risky investments.*” This sentence is ambiguous because either the grandmother or the heiress could be interpreted as the person who bankrupted herself. However, in these globally ambiguous sentences, the structural representation of the sentence does not alert the reader to that ambiguity (i.e., both interpretations are “correct” without additional context) and thus should not require resolution. It is possible, therefore, that the reader would not realize the potential secondary interpretation and would instead accept the interpretation that they believe to be the most sensible as they read (Ferreira & Patson, 2007). Since it is the restructuring process that should underlie the relationship between garden path sentence comprehension and creative problem solving, the comprehension of such sentences should not relate to creative problem solving as there is no need for the reader to restructure their understanding. However, should the reader be alerted to the global ambiguity (e.g., through context), one would expect restructuring processes to take place, and such correlations to return. With the addition of an analytic problem-solving condition, multiple forms of ambiguous sentences, and two individual difference variables, Experiment II uses hierarchical linear regression to test whether ambiguous sentence comprehension relates to performance on different problem-solving tasks, after controlling for individual difference variables and routine language comprehension skills.

#### 3.2.1. Method

**Participants.** Ninety-seven undergraduate students (32 males, 65 females) enrolled in General Psychology at Mississippi State University who had not participated in the previous experiment participated in Experiment II. All students were native English speakers and received course credit upon completion of the experiment. The number of participants was again chosen via a power analysis with the goal of achieving a power of 0.80 for detecting medium-to-large effect sizes using multiple linear regression with three predictor variables (Cohen, 1992) and represented a convenience sample. This study was reviewed and approved by the Mississippi State University IRB. All participants volunteered and provided written consent to participate.

**Materials.** Participants completed five tasks, all of which were presented on desktop computers, with the total running time for all tasks lasting up to an hour and a half. In addition to a more elaborate sentences task, participants completed a creative problem-solving task (the anagrams task), an analytic problem-solving task (the modular arithmetic task), a gF task (figural analogies), and a complex WMC span task (symmetry span). Participants completed each task in the fixed order described, and items within each task were randomized and varied in difficulty. Accuracy and response times were recorded for all items in each task.

***The Sentences Task*.** The sentences task in Experiment II included both locally and globally ambiguous sentences, as well as control sentences matched in structure to each of these two types of ambiguity (see Table 4 for examples). Locally ambiguous and locally non-ambiguous sentence comprehension were assessed using an abbreviated Garden Path Task from Experiment I—participants provided truth-value judgments for 12 locally ambiguous garden path sentences and a randomly chosen, counterbalanced subset (24) of the previously used non-ambiguous control sentences (garden path control). Comprehension was assessed using the same truth-value statements and judgment process used in Experiment I.

Also embedded within the sentences task were 12 globally ambiguous sentences, as measured by relative clause (RC) ambiguous sentences (adapted from Swets et al., 2007), in which a relative clause can correctly attach to one of two preceding noun phrases and thus result in two correct, but different, interpretations of the sentence. In accordance with the good-enough model of comprehension, readers should therefore consider their initial interpretations of these RC ambiguous sentences acceptable and should not require resolution processes, as locally ambiguous garden path sentences do. Finally, a set of non-ambiguous control sentences (24) matched in structure to the RC ambiguous sentences were also included. For these RC control sentences, a verb phrase was presented between two noun phrases before the relative clause, making it clear which of the two noun phrases the following relative clause referenced.

Because the ambiguous RC sentences maintained two viable interpretations, the truth-value comprehension measure used for locally ambiguous sentences would be inappropriate. Therefore, after reading and fully comprehending a sentence of this type, participants were shown a multiple-choice style statement with three response options from which to choose the correct answer. Six of these multiple-choice statements asked about which noun phrase the relative clause should attach to, with the first two options referring to each of the two noun phrases and the third option suggesting that both of the first two options could be correct. For example, for the sentence, “*The sister of the schoolgirl who burned herself the other day was usually very careful*,” the comprehension measure stated “*___ burned herself the other day? 1. Sister 2. Schoolgirl 3. Both 1 and 2 could be correct.*” For these six “RC-True” ambiguous sentences, the third option was considered the correct answer, as the relative clause could relate to either the sister or the schoolgirl. The remaining six multiple-choice statements provided a counterbalanced condition, in which the comprehension measure asked which noun phrase a non-ambiguous part of the sentence—the final verb phrase—referenced. For example, for the sentence, “*The sister of the actress who shot herself on the balcony was under investigation*,” the comprehension measure was “*___ was under investigation? 1. The sister 2. The actress 3. Both 1 and 2 could be correct.*” For these six “RC-NP1” ambiguous sentences, the multiple-choice statements emphasized the role of the first noun phrase, so the first option was considered the correct answer for these sentences. Counterbalancing the comprehension measure in this way therefore allowed for a better understanding of a person’s ability to not only notice true global ambiguities (RC-True) but also to correctly attach non-ambiguous parts of the globally ambiguous sentences (RC-NP1), providing a robust measure of a person’s ability to appropriately grapple with the multiple meanings within a globally ambiguous sentence. Accuracies on all 12 of these sentences were combined into a single score and were analyzed together for this reason. For the 24 RC control sentences, the multiple-choice questions were counterbalanced, such that half of the questions referred to the first noun phrase and the other half referred to the second noun phrase.

In all, participants viewed 72 test sentences, divided among four conditions: locally ambiguous garden path sentences (12), globally ambiguous RC sentences (12), non-ambiguous garden path control sentences (24), and non-ambiguous RC control sentences (24). Items within all four conditions were randomized in the sentences task, so participants were prompted to respond differently for the garden path sentences than for the RC sentences. Participants viewed and responded to five example sentences (one garden path, two garden path control, one RC ambiguous, and one RC control) before starting the task to familiarize themselves with the procedure.

***The Anagram Task*.** The anagram task’s materials and procedure were identical to that in Experiment I, with the exception of correcting an item with a typo.

***The Modular Arithmetic Task.*** The modular arithmetic task used adapted materials and procedures from Beilock and DeCaro (2007). The modular arithmetic task assesses mathematical problem-solving ability and is novel to most participants. The goal of the task is to determine whether the remainder of a modular equation (e.g., 42 = 20(*mod* 11)) is a whole number. A simple algorithm can be used to derive the solution and make a validity judgment: first, the second number should be subtracted from the left-hand side (42 − 20 = 22); next, the difference from the first step should be divided by the modular number (22/11 = 2). If the remainder is a whole number, as in the example provided, the participant would respond with “T” for “true.” For equations resulting in remainders (e.g., 59 = 15(*mod* 8) = 5.5), participants would respond with “F” for “false.” Problems represented a range of difficulty and were counterbalanced for validity judgments. Participants solved six practice problems and 48 test problems and were scored for the proportion of correct items.

***The Figural Analogies Task*.** The figural analogies task used materials and procedures from Lohman and Hagen (2001) and served as a measure of gF for this study. Participants solved 25 test problems of the form “A is to B as C is to ___,” where A and C are figures of relatively simple shapes that undergo various transformation rules (e.g., rotation, flip, change in size, etc.) to produce B and D. The solver must determine the transformation rule that is demonstrated by A:B and apply it to the relationship between C and the unknown figure. Participants then chose the correct answer from five possible response options presented in a response bank next to the problem. Participants had 10 min to solve as many of the 25 problems as possible before the task timed out.

***The Symmetry Span Task*.** The symmetry span task used materials and procedures from Unsworth et al. (2009). The task consisted of two intervening components within a single trial: a processing task and a memory task. The processing task required participants to determine whether a pattern of shaded squares in an eight-by-eight array was vertically symmetrical. Afterwards, the memory task required participants to remember a single shaded square in a four-by-four array. At the end of a trial, which ranged between two and five items, participants clicked the to-be-remembered squares in a blank four-by-four array in the correct serial order in which they were presented. The entire task consisted of three iterations of each trial length. The task was scored as the average proportion of squares recalled in the correct serial position (regardless of item size) across all trials (i.e., partial-credit unit scoring; Conway et al., 2005).

**Procedure.** Participants were seated in a testing room at a desktop computer. After providing written informed consent, the experimenter provided participants with general instructions about the tasks to be completed. The experimenter also loaded and started each of the tasks for every participant. Upon completion of the experiment, participants were individually thanked, debriefed, and dismissed.

#### 3.2.2. Results

**Initial Analyses.** See Table 5 for descriptive statistics and Table 6 for Pearson’s correlations of task accuracy. Accuracy on the anagrams task and the figural analogies task significantly correlated with all tasks administered, including each of the four sentence types in the sentences task. Modular arithmetic significantly correlated with all tasks except symmetry span and all sentence types except for garden path sentences. Symmetry span did not significantly correlate with any tasks except for figural analogies and anagrams. Once again, there was lower reliability for garden path sentences (both control and ambiguous versions). This may suppress correlations with those variables.

Initial analyses indicated that garden path and RC sentences behaved similarly. Table 6 demonstrates that the correlations between garden path and RC sentences were comparable in predicting anagram accuracy. Though unexpected, this may be attributable to the way comprehension was assessed for RC sentences. It is likely that the third response option on the RC comprehension measure (i.e., “Both a and b could be correct”) highlighted the fact that two interpretations of a sentence were possible. This in turn altered participants’ reading strategies, forcing them to restructure rather than accept their initial interpretation to make the most well-informed response. Because participants seemed to restructure on RC sentences, garden path and RC sentence comprehension scores were normalized and averaged into a composite ambiguous sentence comprehension score for each person. Similarly, garden path control and RC control sentences were combined into a composite control sentence comprehension score. For simplicity of reporting results, analyses will focus on these composite sentence comprehension scores. However, it is worth noting that these variables were combined primarily as a data-reduction technique, to provide simplified analyses that may be easier to interpret. A major concern is that these measures do, in fact, interact differently with problem-solving. As such, abbreviated analyses separating by sentence type are highlighted and briefly discussed as well.

**Creative Problem Solving.** As a manipulation check, a partial correlation between anagram accuracy and composite ambiguous sentence accuracy was conducted, controlling for modular arithmetic performance. When controlling for modular arithmetic performance, anagram accuracy significantly correlated with composite ambiguous sentence accuracy, *r*(95) = 0.41, *p* < .001. When correlating ambiguous sentence comprehension with modular arithmetic performance and controlling for anagram accuracy, there was no significant correlation, *r*(95) = 0.05, *p* = .66. This suggests that anagram performance is indeed measuring variance related to ambiguous sentence comprehension, over and above performance on a standard analytic problem-solving task.

To address the question of whether ambiguous sentences uniquely predict creative problem solving, two hierarchical linear regressions were conducted with anagram performance as the dependent variable. The first model included WMC in the first step, and the second model included gF in the first step. Composite control and ambiguous sentences were entered into the second and third steps (respectively) for both models. This ordering of variables provides the strictest test of the hypotheses, in that the general constructs’ variance is accounted for first, followed by the control variable, and only then is the variable of interest entered into the equation. Thus, a significant R^2^ change represents significant variance being explained by ambiguous sentence processing after controlling for all other variables. The results of the first model, which accounted for WMC in the first step, are presented in Table 7. The results of the second model, which accounted for gF in the first step, are presented in Table 8. VIF indices of all variables in each model were less than 1.5, suggesting no issues due to multicollinearity.

As Table 7 and Table 8 demonstrate, after controlling for a person’s WMC and propensity for understanding non-ambiguous sentences, the ability to recognize and overcome ambiguity was a unique predictor of creative problem solving, explaining 9% additional variance in the model (ΔR^2^ = 0.09; a moderate increase). Similarly, when controlling for a person’s gF and ability to understand non-ambiguous sentences, the ability to recognize and overcome ambiguity was uniquely predictive of creative problem solving, explaining 5% additional variance (ΔR^2^ = 0.05; a small increase).

Variants of these models were re-run with the composite sentence variables separated by sentence type (garden path and RC), and the results mirror those of the composite models almost exactly. Indeed, both garden path sentence resolution (ΔR^2^ = 0.06, B = 0.26, *t* (96) = 2.64, *p* = .010, 95% CI = [0.064, 0.458]) and RC sentence comprehension (ΔR^2^ = 0.10, B = 0.90, *t* (96) = 3.48, *p* = .001, 95% CI = [0.388, 1.416]) uniquely predicted creative problem-solving performance, even after accounting for WMC and the ability to understand the control sentences of each type. Similarly, RC sentence comprehension uniquely predicted creative problem-solving performance, after accounting for gF and RC control sentences (ΔR^2^ = 0.05, B = 0.66, *t* (96) = 2.60, *p* = .011, 95% CI = [0.156, 1.162]). However, garden path sentence resolution was no longer a unique predictor of creative problem solving when accounting for gF and garden path control sentences (ΔR^2^ = 0.02, B = 0.18, *t* (96) = 1.83, *p* = .070, 95% CI = [−0.015, 0.369]).

To further elucidate these results, models were re-run with RC-True sentence accuracy (which required addressing ambiguity to respond) or RC-NP1 sentence accuracy (which did not) entered in the final step. When accounting first for WMC and then RC control accuracy, RC-True sentences predicted unique variance in creative problem-solving (ΔR^2^ = 0.05, B = 0.23, *t* (96) = 2.42, *p* = .017, 95% CI = [0.040, 0.409]) but RC-NP1 sentences did not (ΔR^2^ = 0.00, B = −0.007, *t* (96) = −0.08, *p* = .941, 95% CI = [−0.203, 0.189]). The same result was found when accounting for gF: RC-True sentences predicted unique variance in creative problem-solving (ΔR^2^ = 0.03, B = 0.17, *t* (96) = 2.02, *p* = .046, 95% CI = [0.003, 0.345]), but RC-NP1 sentences did not (ΔR^2^ = 0.001, B = −0.03, *t* (96) = −0.30, *p* = .765, 95% CI = [−0.207, 0.153]).

**Analytic Problem-Solving.** Although the models above demonstrate that ambiguous sentence comprehension uniquely predicts creative problem solving even after accounting for individual difference variables known to be important for reading comprehension, it is necessary to demonstrate that ambiguous sentence comprehension is not predictive of *all* types of problem solving. Because restructuring should not be involved in routine, analytic problem solving (DeCaro et al., 2016; Fleck, 2008; Lavric et al., 2000; Schooler et al., 1993; Wiley and Jarosz, 2012), the data must also be able to demonstrate that ambiguous sentence comprehension is predictive of *only* creative problem solving and not of analytic problem solving. Thus, two hierarchical linear regressions were again conducted, but with modular arithmetic performance as the dependent variable, and with composite control sentence comprehension and composite ambiguous sentence comprehension as the second and third steps (respectively). Congruent with the prior analyses, the first model included WMC in the first step, and the second model included gF in the first step. The results of the first model, which accounted for WMC in the first step, are presented in Table 9. The results of the second model, which accounted for gF in the first step, are presented in Table 10.

As Table 9 and Table 10 demonstrate, after controlling for a person’s WMC and propensity for understanding non-ambiguous sentences, the ability to recognize and overcome ambiguity was not predictive of analytic problem solving. Similarly, when controlling for a person’s gF and ability to understand non-ambiguous sentences, the ability to recognize and overcome ambiguities was again not predictive of analytic problem solving. In both cases, the ΔR^2^ was less than 0.01, suggesting virtually no additional variance was explained. Because the hypotheses relating to these analyses predicted a null result, a Bayesian analysis was also conducted. WMC and control sentence accuracy were added to the null model predicting modular arithmetic performance, with the final model adding in the composite ambiguous sentence comprehension variable. Results indicated that the data were 3.31 times more likely under the null model (BF_10_ = 0.30). A similar analysis with gF also supported the null model as 3.45 times more likely (BF_10_ = 0.29).

Variants of these models were re-run with the ambiguous and control sentences separated by sentence type (garden path and RC), and the results mirror those of the composite models exactly. Indeed, neither garden path sentence resolution (ΔR^2^ = 0.00, B = 0.01, *t* (96) = 0.12, *p* = .904, 95% CI = [−0.205, 0.232]) nor RC sentence comprehension (ΔR^2^ = 0.02, B = 0.41, *t* (96) = 1.47, *p* = .146, 95% CI = [−0.144, 0.956]) uniquely predicted analytic problem-solving performance, after accounting for WMC and the ability to understand the control sentences of each type. Similarly, neither garden path sentence resolution (ΔR^2^ = 0.003, B = −0.06, *t* (96) = −0.58, *p* = .562, 95% CI = [−0.273, 0.149]) nor RC sentence comprehension (ΔR^2^ = 0.004, B = 0.20, *t* (96) = 0.73, *p* = .470, 95% CI = [−0.347, 0.746]) uniquely predicted analytic problem-solving performance, after accounting for gF and control sentence comprehension of both types. Together, these findings demonstrate that overcoming ambiguities in language are uniquely related to creative problem solving but not analytic problem solving.

Models were again re-run with RC-True sentences or RC-NP1 sentences entered in the final step. When accounting for WMC and RC control sentences, RC-True sentences predicted unique variance in modular arithmetic performance (ΔR^2^ = 0.05, B = 0.22, *t* (96) = 2.35, *p* = .021, 95% CI = [0.034, 0.409]), but RC-NP1 sentences did not (ΔR^2^ = 0.02, B = −0.13, *t* (96) = −1.32, *p* = .189, 95% CI = [−0.329, 0.066]). The same result was found when accounting for gF: RC-True sentences predicted unique variance in analytic problem solving (ΔR^2^ = 0.04, B = 0.19, *t* (96) = 2.09, *p* = .039, 95% CI = [0.009, 0.368]) but RC-NP1 sentences did not (ΔR^2^ = 0.02, B = −0.15, *t* (96) = −1.63, *p* = .107, 95% CI = [−0.339, 0.034]).

**Combined WMC and gF.** A final analysis repeated the earlier analyses, but combined WMC and gF in the first step simultaneously as a stronger test of the current predictions. As noted in Table 11 and Table 12, gF (but not WMC) predicted both creative and analytic problem solving, as did control sentence performance. However, adding ambiguous sentence performance to the model only improved model fit when predicting creative problem solving, and not analytic problem solving. A Bayesian model with WMC, gF, and control sentence processing predicting analytic problem solving as the null model, and adding ambiguous sentence processing in the final model, suggested the data were 2.91 times more likely under the null model (BF_10_ = 0.34).

**Exploratory Analyses.** As in Experiment I, response time analyses were conducted (though no a priori hypotheses were made regarding the results). For both garden path and RC sentences, combined WMC and gF models were conducted to see if results replicated findings from the accuracy analyses. In models predicting anagram response time, garden path sentence reading time did not improve model fit over and above a model with WMC scores, gF scores, and control sentence reading time (ΔR^2^ = 0.03, *p* = .05). The same was true for a model using RC reading time in lieu of garden path sentence reading time, ΔR^2^ = 0.001, *p* = .70. When predicting modular arithmetic performance, adding garden path sentences to the model improved model fit over and above a model with WMC scores, gF scores, and control sentence reading time (ΔR^2^ = 0.09, *p* = .003). In the final model, control sentence reading speed negatively related to solution speed on modular arithmetic problems, β = −0.28, *p* = .046, while garden path sentence reading time related positively, β = 0.44, *p* = .003. Using RC reading time in lieu of garden path sentence reading time led to a null result, ΔR^2^ = 0.002, *p* = .67.

A final exploratory analysis conceptually replicated the analyses in Table 11 and Table 12. Using anagram accuracy as an outcome, and utilizing WMC, gF, and garden path ambiguous and control sentence accuracy as predictors, the analysis was repeated with the inclusion of garden path ambiguous sentence-processing time as a control variable. As seen in Table 13, including garden path sentence response time as a control variable resulted in the same pattern of results witnessed previously, with ambiguous garden path sentence accuracy predicting anagram solution over and above all other variables. In contrast, as seen in Table 14, this pattern does not appear for modular arithmetic performance.

## 4. General Discussion

These two studies are consistent with restructuring during creative problem solving drawing upon the same processes as the revisions necessary to understand ambiguous language. Experiment I demonstrated that creative problem solving and garden path sentence comprehension are uniquely related beyond what can be explained by non-ambiguous sentence comprehension. Experiment II further established that this relationship cannot be explained by WMC or gF, nor does it exist with analytic problem solving. As in Experiment I, the lack of a relationship with analytic problem solving does not necessarily provide evidence of no relationship existing. However, the Bayes factors consistently demonstrated roughly three times the evidence in favor of the null hypothesis over the alternative, providing weak-to-positive support in favor of the lack of a relationship between analytic problem solving and garden path sentence processing (Jarosz & Wiley, 2014). Further, when considering creative problem solving, there were small-to-medium effect size increases in model fit with the inclusion of ambiguous sentence processing accompanied by standardized betas demonstrating that ambiguous sentence processing explains almost as much as, or more, unique variance than gF and WMC, respectively.

Other analyses from Experiment II investigated the independent effects of ambiguous sentence types on problem solving, demonstrating patterns congruent with the combined sentence-processing results. In almost every case, ambiguous sentence processing predicted creative problem-solving over and above WMC and gF. Even for the one exception, in which garden path sentences did not predict creative problem solving above gF, results trended in the same direction; this null effect may be attributable to the lower reliability of both the garden path and garden path control sentences suppressing its predictive ability when compared to the other, more reliable sentence types and predictors.

Exploratory response time analyses were inconsistent in their results, particularly in Experiment II. In only one case (using ambiguous GPS reading time to predict modular arithmetic solution time) was there a significant improvement in model fit after adding GPS processing to the model. In this case, faster control sentence reading related to slower solution times, while faster ambiguous sentence reading related to faster solution times. Given the lack of a priori hypotheses, these findings are difficult to interpret. However, in a mixed accuracy and response time analysis, controlling for the reading time on ambiguous GPS did not prevent ambiguous GPS accuracy from uniquely explaining anagram accuracy. This continues to support the idea of a restructuring mechanism.

Importantly, it should be noted that there are no direct measures of restructuring in this study. The pattern of results is consistent with what would be expected giving a shared restructuring mechanism, particularly given the prediction of creative, but not analytic, problem-solving measures. However, without direct measures, the present findings remain an initial step towards demonstrating a restructuring mechanism.

Reliability was low for both the GPS ambiguous (at α = 0.60) and control (α = 0.40) sentences. This could have a concerning impact on results, particularly as low reliability could decrease correlation and regression coefficients, and lead to increased estimates of effect size. However, there are several ways to alleviate these concerns. First, there were a number of significant findings using the ambiguous GPS sentences, both alone and in concert with RC sentences, suggesting that in the case of creative problem solving this concern is not warranted. It seems highly unlikely that this (still fairly reliable) variable demonstrated suppressed regression coefficients only in cases where it was predicting analytic problem-solving, while not suppressing them for creative problems. More concerning is the very low reliability of the GPS control sentences in Experiment II. Here it should be noted that even in Experiment I, where the reliability was much higher (at α = 0.74), control sentence accuracy could not account for ambiguous sentence processing’s relationship to creative problem solving. Further, Experiment II contained measures of WMC and gF, both of which fulfill a similar role as control sentence processing, and neither of which could account for ambiguous sentence processing’s ability to predict creative performance.

Though it was initially unexpected that RC-True sentence performance would explain creative problem-solving performance, the nature of the accuracy measure may have impacted results. Specifically, providing a “both/and” option may force participants to note and resolve the sentence’s ambiguity. This is supported by the failure of RC-NP1 sentences—in which the multiple-choice questions did not reference the sentence’s ambiguity—to uniquely predict creative problem-solving. Thus, only when ambiguity required resolution was there a relationship between ambiguous sentence processing and creative problem solving. The “both/and” nature of RC-True sentences may also inform why they were the only sentence type to explain unique variance in analytic problem-solving performance. Just as creative problems can be solved via creative or analytic processes (Kounios & Beeman, 2014), artificially forcing readers to address ambiguity could have led to multiple methods of interpretation: sometimes via restructuring, and sometimes via a more analytic approach. To be clear, this is a significant limitation that warrants further research. Future work should assess ambiguity resolution in RC sentences using a different methodology to avoid changing participant strategies. Promising candidates may be using eye tracking or a moving window paradigm to look at word-level processing. Despite this, the differences between RC-True and RC-NP1 sentences, as well as the consistent findings with GPS sentences, provide initial (though not perfect) evidence consistent with a restructuring mechanism.

One concern is that the sentence-processing tasks required two different forms of response: a truth value judgment, and a multiple-choice selection, depending on whether the item was a garden path sentence or if it used relative clause ambiguity. It is possible that the decision to use two response formats may have introduced measurement confounds. That being said, there are two reasons why these differences in methodology were beneficial. First, truth-value statements would not have been appropriate for the RC sentences. Using a varying response methodology allowed for a more accurate assessment of participants’ reading. Second, the use of two different methodologies ultimately reduced the similarity of surface features in the two sentence types, reducing the potential impact of task-specific variance in the composite variable.

It is also unclear whether restructuring processes play a role in language comprehension beyond single sentences. For example, narrative texts may force readers to reassess prominent aspects of a story, requiring the inclusion of formerly “irrelevant” details to understand the characters’ situation or motivation (Anderson & Pichert, 1978). Thus, the reader of a detective novel may experience a rush analogous to insight upon realizing that a once minor detail is key to unlocking a murder mystery (Auble et al., 1979). This “restructuring” of the situation model may draw upon the same processes demonstrated here. It is similarly unclear whether these restructuring processes would be utilized outside of a college population, such as in developing or aging readers.

It is worth noting that both studies utilized an undergraduate student population from a university in the southern United States. As such, the population was one of young, native-English speakers. If the above findings are due to quirks of the English language, or are driven by specific aspects of culture, the findings above may not generalize to a wider population. In the case of differing educational backgrounds, there is little reason to expect that these results would not hold true. Ambiguity and its resolution are fixtures in almost every aspect of life. However, these particular results may depend somewhat on the peculiarities of language. Most languages have some aspect of ambiguity, but this ambiguity will take different forms depending on the nuances of the semantics and syntax that are present. To that end, this study could likely not be replicated by simply translating sentences to another language. That said, future research could test these findings utilizing aspects of ambiguity in other languages (or indeed, using non-linguistic measures of ambiguity resolution).

The present studies provide initial evidence for a novel, shared cognitive process that initiates restructuring or revisions to ambiguous information across multiple contexts. Though merely a first step, this work provides an understanding of how individuals grapple with inconsistencies between the goals of their tasks and their representations of the world. Future work should not only address the role of restructuring beyond sentences and creative problems, but also explore how this mechanism may apply in any context in which ambiguities must be revised, reordered, or restructured.

## Figures and Tables

**Table 1:** Garden path task examples.

Type	Example Sentence	Truth-Value Statement	Answer
GP	When the actor performs the play containing profanities runs late and people leave.	People left the play that contained profanities.	T
GP	While the artist drew the child standing on the sidewalk gobbled up his ice cream.	The artist drew the child eating ice cream.	F
Cont	While the ship in the bay sank the band played a mournful tune.	The band sank with the ship.	F
Cont	As the weather became warmer the majestic trees began growing leaves.	The weather became warmer.	T

*Note.* Type = Sentence type; GP = Garden path sentence; Cont = Control sentence.

**Table 2:** Descriptive statistics and Cronbach’s alpha for task accuracy and sentence types.

Task	*N*	*M*	*SD*	Min	Max	Skew	Kurtosis	α
Rebus	85	0.51	0.15	0.08	0.79	−0.72	0.39	0.73
Anagram	85	0.53	0.14	0.13	0.90	−0.11	0.12	0.72
CreatComp	85	0.00	0.84	−1.82	1.93	−0.23	−0.56	-
GP	85	0.68	0.21	0.33	1.00	−0.04	−1.17	0.62
Cont	85	0.85	0.11	0.47	1.00	−1.43	2.25	0.74

*Note.* CreatComp = composite creativity; GP = garden path sentences; Cont = control sentences.

**Table 3:** Pearson’s *r* for task accuracy and sentence types in Experiment I.

	Rebus	Anagram	CreatComp	GP	Cont
Rebus	1	-	-	-	-
Anagram	0.42 *	1	-	-	-
CreatComp	0.84 *	0.84 *	1	-	-
GP	0.42 *	0.27 *	0.41 *	1	-
Cont	0.29 *	0.21	0.28 *	0.41 *	1

*Note.* * *p* < .05 (two-tailed); CreatComp = composite creativity; GP = garden path sentences; Cont = control sentences.

**Table 4:** Sentences task examples.

	Type	Example Sentence	Truth-Value Statement	Answer
Local	GP (12)	When the actor performs the play containing profanities runs late and people leave.	People left the play that contained profanities.	T
	GPcont (24)	While the ship in the bay sank the band played a mournful tune.	The band sank with the ship.	F
Global	RCtrue (6)	The maid of the princess who scratched herself in public was terribly embarrassed.	____ scratched herself in public. 1. maid 2. princess 3. both 1 and 2 could be correct	3
	RC.NP1 (6)	The uncle of the fireman who criticized himself far too often was painting in the bedroom.	____ was painting in the bedroom. 1. uncle 2. fireman 3. both 1 and 2 could be correct	1
	RCcont (24)	The baker amused the customer who stayed late into the evening.	____ stayed late into the evening. 1. baker 2. customer 3. both 1 and 2 could be correct	2

*Note.* Type = sentence type; GP = garden path sentence; GPcont = garden path control sentence; RCtrue = relative clause ambiguous sentence (truth-value statement concerning relative clause attachment); RC.NP1 = relative clause ambiguous sentence (truth-value statement concerning the first noun phrase); RCcont = relative clause control sentence.

**Table 5:** Descriptive statistics.

Task	*N*	*M*	*SD*	Min	Max	Skew	Kurtosis	α
GP	97	0.64	0.21	0.25	1.00	0.231	−1.133	0.60
GPcont	97	0.85	0.09	0.63	1.00	−0.834	0.059	0.40
RC	97	0.47	0.15	0.08	1.00	0.758	2.990	0.89
RCcont	97	0.89	0.12	0.46	1.00	−1.722	2.821	0.78
Anagrams	97	0.60	0.17	0.17	0.93	−0.666	0.357	0.81
ModArith	97	0.70	0.13	0.38	0.94	−0.380	−0.461	0.79
FigAnalogy	97	0.56	0.19	0.12	0.89	−0.285	−0.545	0.79
SymmSpan	97	0.67	0.17	0.25	1.00	−0.214	−0.428	0.78

*Note.* GP = garden path sentences; GPcont = garden path control sentences; RC = relative clause sentences; RCcont = relative clause control sentences.

**Table 6:** Pearson’s *r* for task accuracy and sentence types in Experiment II.

	GP	GPcont	RC	RCcont	Ana	ModAr	FigAna	Symm
GP	1	-	-	-	-	-	-	-
GPcont	0.45 *	1	-	-	-	-	-	-
RC	0.39 *	0.27 *	1	-	-	-	-	-
RCcont	0.32 *	0.43 *	0.24 *	1	-	-	-	-
Anagrams	0.38 *	0.39 *	0.39 *	0.35 *	1	-	-	-
ModArith	0.13	0.29 *	0.21 *	0.35 *	0.32 *	1	-	-
FigAnalogy	0.33 *	0.38 *	0.33 *	0.35 *	0.51 *	0.41 *	1	-
SymmSpan	−0.009	0.04	0.11	0.19	0.26 *	0.15	0.28 *	1

*Note.* * *p* < .05 (two-tailed). GP = garden path sentences; GPcont = garden path control sentences; RC = relative clause sentences; RCcont = relative clause control sentences.

**Table 7:** Effects of WMC, control sentences, and ambiguous sentences on creative problem-solving.

		R^2^	*F*	ΔR^2^	Δ*F*	B	SE	β	*t*	*p*	95% CI for B
Step 1	0.07	6.81	**0.07**	6.81 *						
	**WMC**					**0.26**	**0.10**	**0.26**	**2.61**	**0.011**	**[0.062, 0.455]**
Step 2	0.24	14.42	**0.17**	20.62 *						
	WMC					0.20	0.09	0.20	2.22	0.029	[0.021, 0.383]
	**Control**					**0.49**	**0.11**	**0.41**	**4.54**	**0.000**	**[0.276, 0.704]**
Step 3	0.32	14.67	**0.09**	11.84 *						
	WMC					0.21	0.09	0.21	2.44	0.017	[0.039, 0.381]
	Control					0.31	0.12	0.26	2.67	0.009	[0.079, 0.536]
	**Ambiguous**					**0.70**	**0.20**	**0.33**	**3.44**	**0.001**	**[0.295, 1.100]**

*Note.* Each model is compared to the prior model(s). * *p* < .05 (two-tailed).

**Table 8:** Effects of gF, control sentences, and ambiguous sentences on creative problem-solving.

		R^2^	*F*	ΔR^2^	Δ*F*	B	SE	β	*t*	*p*	95% CI for B
Step 1	0.26	32.96	**0.26**	32.96 *						
	**gF**					**0.51**	**0.09**	**0.51**	**5.74**	**0.000**	**[0.332, 0.683]**
Step 2	0.32	21.88	**0.06**	8.28 *						
	gF					0.39	0.10	0.39	4.12	0.000	[0.201, 0.577]
	**Control**					**0.32**	**0.11**	**0.27**	**2.88**	**0.005**	**[0.100, 0.545]**
Step 3	0.36	17.63	**0.05**	6.54 *						
	gF					0.33	0.10	0.33	3.51	0.001	[0.144, 0.520]
	Control					0.22	0.12	0.18	1.87	0.065	[−0.014, 0.449]
	**Ambiguous**					**0.52**	**0.20**	**0.25**	**2.56**	**0.012**	**[0.116, 0.918]**

*Note.* Each model is compared to the prior model(s). * *p* < .05 (two-tailed).

**Table 9:** Effects of WMC, control sentences, and ambiguous sentences on analytic problem-solving.

		R^2^	*F*	ΔR^2^	Δ*F*	B	SE	β	*t*	*p*	95% CI for B
Step 1	0.02	2.29	0.02	2.29						
	WMC					0.15	0.10	0.15	1.51	0.134	[−0.048, 0.355]
Step 2	0.15	8.54	**0.13**	14.48 *						
	WMC					0.10	0.10	0.10	1.08	0.284	[−0.087, 0.293]
	**Control**					**0.43**	**0.11**	**0.36**	**3.81**	**0.000**	**[0.206, 0.657]**
Step 3	0.16	5.69	0.001	0.14						
	WMC					0.10	0.10	0.10	1.08	0.282	[−0.087, 0.295]
	Control					0.41	0.13	0.35	3.19	0.002	[0.155, 0.665]
	Ambiguous					0.08	0.23	0.04	0.37	0.711	[−0.365, 0.533]

*Note.* Each model is compared to the prior model(s). * *p* < .05 (two-tailed).

**Table 10:** Effects of gF, control sentences, and ambiguous sentences on analytic problem-solving.

		R^2^	*F*	ΔR^2^	Δ*F*	B	SE	β	*t*	*p*	95% CI for B
Step 1	0.17	18.81	**0.17**	18.81 *						
	gF					**0.41**	**0.09**	**0.41**	**4.34**	**0.000**	**[0.220, 0.593]**
Step 2	0.22	12.92	**0.05**	6.03 *						
	gF					0.30	0.10	0.30	2.94	0.004	[0.097, 0.500]
	Control					**0.30**	**0.12**	**0.25**	**2.46**	**0.016**	**[0.056, 0.533]**
Step 3	0.22	8.57	0.001	0.12						
	gF					0.31	0.11	0.31	2.93	0.004	[0.099, 0.515]
	Control					0.31	0.13	0.26	2.41	0.018	[0.055, 0.567]
	Ambiguous					−0.077	0.22	−0.04	−0.35	0.730	[−0.522, 0.367]

*Note.* Each model is compared to the prior model(s). * *p* < .05 (two-tailed).

**Table 11:** Effects of WMC, gF, control sentences, and ambiguous sentences on creative problem-solving.

		R^2^	*F*	ΔR^2^	Δ*F*	B	SE	β	*t*	*p*	95% CI for B
Step 1	0.27	17.61	**0.27**	17.61 *						
	WMC					0.003	0.002	0.12	1.39	0.17	[−0.001, 0.008]
	**gF**					**0.41**	**0.08**	**0.47**	**5.15**	**0.000**	[0.254, 0.573]
Step 2	0.33	15.15	**0.06**	7.72 *						
	WMC					0.003	0.002	0.12	1.34	0.18	[−0.001, 0.007]
	gF					0.32	0.08	0.36	3.73	0.000	[0.149, 0.486]
	**Control**					**0.47**	**0.17**	**0.26**	**2.78**	**0.007**	[0.136, 0.819]
Step 3	0.38	13.92	**0.05**	7.19 *						
	WMC					0.004	0.002	0.15	1.79	0.08	[−0.000, 0.008]
	gF					0.28	0.08	0.32	3.41	0.001	[0.119, 0.449]
	Control					0.41	0.17	0.23	2.47	0.02	[0.081, 0.749]
	**Ambiguous**					**0.17**	**0.06**	**0.23**	**2.68**	**0.009**	**[0.043, 0.288]**

*Note.* Each model is compared to the prior model(s). * *p* < .05 (two-tailed).

**Table 12:** Effects of WMC, gF, control sentences, and ambiguous sentences on analytic problem-solving.

		R^2^	*F*	ΔR^2^	Δ*F*	B	SE	β	*t*	*p*	95% CI for B
Step 1	0.17	9.43	0.17	9.43 *						
	WMC					0.001	0.002	0.04	0.44	0.66	[−0.003, 0.005]
	**gF**					**0.28**	**0.07**	**0.39**	**4.03**	**0.000**	**[0.140, 0.411]**
Step 2	0.22	8.73	**0.05**	6.27 *						
	WMC					0.001	0.002	0.04	0.37	0.71	[−0.003, 0.004]
	gF					0.20	0.07	0.29	2.75	0.007	[0.056, 0.346]
	**Control**					**0.37**	**0.15**	**0.25**	**2.50**	**0.01**	**[0.076, 0.663]**
Step 3	0.23	7.04	0.01	1.75						
	WMC					0.001	0.002	0.05	0.57	0.57	[−0.003, 0.005]
	gF					0.19	0.07	0.27	2.53	0.01	[0.040, 0.332]
	Control					0.34	0.15	0.24	2.31	0.02	[0.047, 0.637]
	Ambiguous					0.07	0.05	0.13	1.32	0.19	[−0.036, 0.180]

*Note.* Each model is compared to the prior model(s). * *p* < .05 (two-tailed).

**Table 13:** Effects of WMC, gF, control sentences, and ambiguous sentence accuracy and reading time on creative problem solving.

		R^2^	*F*	ΔR^2^	Δ*F*	B	SE	β	*t*	*p*	95% CI for B
Step 1	**0.27**	**17.61**	**0.27**	17.61 *						
	**WMC**					**0.47**	**0.09**	**0.47**	**5.15**	**0.000**	**[0.290, 0.654]**
	gF					0.13	0.09	0.13	1.39	0.17	[−0.055, 0.309]
Step 2	0.30	13.16	0.03	3.38						
	WMC					0.41	0.09	0.41	4.20	0.08	[0.215, 0.600]
	gF					0.17	0.09	0.17	1.81	0.000	[−0.016, 0.354]
	GP RT					0.00	0.00	0.17	1.84	0.07	[−0.000, 0.000]
Step 3	**0.33**	**11.30**	**0.03**	4.30 *						
	WMC					0.36	0.09	0.36	3.61	0.000	[0.160, 0.552]
	gF					0.16	0.09	0.16	1.78	0.08	[−0.019, 0.345]
	GP RT					0.00	0.00	0.09	0.87	0.39	[−0.000, 0.000]
	**Control**					**2.34**	**1.13**	**0.21**	**2.07**	**0.04**	**[0.098, 4.59]**
Step 4	**0.36**	**10.21**	**0.03**	4.26 *						
	WMC					0.31	0.09	0.31	3.15	0.002	[0.115, 0.509]
	gF					0.18	0.09	0.18	2.01	0.048	[0.002, 0.361]
	GP RT					0.00	0.00	0.10	0.99	0.32	[−0.000, 0.000]
	Control					1.47	1.18	0.13	1.24	0.22	[−0.888, 3.83]
	**Ambiguous**					**0.96**	**0.47**	**0.20**	**2.06**	**0.04**	**[0.036, 1.89]**

*Note.* Each model is compared to the prior model(s). * *p* < .05 (two-tailed).

**Table 14:** Effects of WMC, gF, control sentences, and ambiguous sentence accuracy and reading time on analytic problem-solving.

		R^2^	*F*	ΔR^2^	Δ*F*	B	SE	β	*t*	*p*	95% CI for B
Step 1	**0.17**	**9.43**	**0.17**	9.43 *						
	**WMC**					**0.05**	**0.01**	**0.39**	**4.03**	**0.000**	**[0.026, 0.078]**
	gF					0.01	0.01	0.04	0.44	0.66	[−0.020, 0.031]
Step 2	0.19	7.36	0.03	2.84						
	WMC					0.04	0.01	0.33	3.18	0.002	[0.016, 0.071]
	gF					0.01	0.01	0.08	0.84	0.40	[−0.015, 0.037]
	GPS RT					0.00	0.00	0.17	1.69	0.10	[−0.000, 0.000]
Step 3	0.20	5.74	0.01	0.92						
	WMC					0.04	0.01	0.30	2.83	0.006	[0.012, 0.068]
	gF					0.01	0.01	0.08	0.81	0.42	[−0.015, 0.037]
	GPS RT					0.00	0.00	0.13	1.15	0.25	[−0.000, 0.000]
	Control					0.15	0.16	0.11	0.96	0.34	[−0.167, 0.479]
Step 4	0.20	4.60	0.00	0.22						
	WMC					0.04	0.01	0.32	2.86	0.005	[0.013, 0.070]
	gF					0.01	0.01	0.08	0.76	0.45	[−0.016, 0.036]
	GPS RT					0.00	0.00	0.12	1.12	0.27	[−0.000, 0.000]
	Control					0.19	0.17	0.13	1.06	0.29	[−0.161, 0.532]
	Ambiguous					−0.03	0.07	−0.05	−0.47	0.64	[−0.169, 0.104]

*Note.* Each model is compared to the prior model(s). * *p* < .05 (two-tailed).

## Data Availability

Neither of the experiments reported in this article were formally preregistered. The data from both studies is available on the Open Science Framework at: https://osf.io/a87k6/overview?view_only=fca6773a1c834df68cc544f1d81c9d9e, accessed on 19 January 2026. The materials used in these studies are widely available, and are available from the authors upon request.
